# Postoperative pneumatocele after right upper lobectomy: a case report

**DOI:** 10.1097/RC9.0000000000001087

**Published:** 2026-10-12

**Authors:** Hironobu Yamaoka, Eri Ota, Kana Nagayama, Ryoji Kawano, Rei Kobayashi

**Affiliations:** Department of Thoracic Surgery, Itabashi Chuo Medical Center, Tokyo, Japan

**Keywords:** case report, lung resection, pneumatocele, thoracoscopy

## Abstract

**Introduction and importance:**

Postoperative pneumatocele is a rare complication after pulmonary resection and may mimic serious conditions such as bronchial stump failure or empyema. Accurate diagnosis is important to avoid unnecessary invasive interventions.

**Case presentation:**

We report a case of a postoperative pneumatocele following a right upper lobectomy in a man in his 60s. On postoperative day 25, he presented with fever, cough, and mild hemoptysis, and computed tomography revealed a large thin-walled cavitary lesion with an air–fluid level. Bronchoscopy showed an intact bronchial stump. A drainage catheter placed for suspected pleural infection was subsequently found to have entered the cystic cavity, resulting in decompression of the lesion. Thoracoscopic exploration demonstrated no empyema cavity and revealed a thin-walled cystic lesion arising from the residual lower lobe. The lesion subsequently regressed to near-complete resolution without additional pulmonary resection.

**Clinical discussion:**

Postoperative pneumatoceles are uncommon and may be difficult to distinguish from bronchial stump failure, empyema, or other postoperative cavitary lesions based on imaging alone. Bronchoscopy and thoracoscopy can facilitate differential diagnosis. In selected patients, cyst decompression and drainage may allow resolution without additional pulmonary resection, once major postoperative complications have been excluded.

**Conclusion:**

Postoperative pneumatocele should be considered in the differential diagnosis of cavitary lesions after lung resection. A stepwise diagnostic approach, including bronchoscopy and thoracoscopy, may help to distinguish this condition from bronchial stump failure and empyema and to avoid unnecessary major surgical intervention.

## Introduction

A pneumatocele is a thin-walled, air-containing cystic lesion typically associated with infection or trauma. Development after pulmonary resection is rare. Early postoperative cavitary lesions require the exclusion of bronchial stump failure, but some may represent a postoperative pneumatocele, for which management ranges from observation to cyst drainage or surgical intervention, depending on the clinical condition.

This case highlights an unusual postoperative complication and emphasizes the importance of careful differential diagnosis of postoperative cavitary lesions.

This case report has been reported in accordance with the SCARE 2025 checklist^[^1^]^.

HIGHLIGHTS

- Postoperative pneumatocele is a rare complication after pulmonary lobectomy.
- Cavitary lesions with an air–fluid level may mimic bronchial stump failure or empyema.
- Bronchoscopy and thoracoscopy can facilitate the differential diagnosis.
- Unintended intralesional drainage resulted in cyst decompression in this case.
- Selected patients may avoid additional pulmonary resection after major complications are excluded.

## Case presentation

A man in his 60s was referred after an abnormal finding on a screening chest radiograph. He had no notable medical history or comorbidities. He had smoked 20 cigarettes per day from age 20 to 36. Chest computed tomography (CT) revealed ground-glass opacities measuring 26 mm in segment 2 and 11 mm in segment one of the right upper lobe. There were no radiographic or physiologic findings suggestive of chronic obstructive pulmonary disease. After interval observation without regression, primary lung cancer was suspected, and video-assisted thoracoscopic right upper lobectomy with systematic lymph node dissection (ND2a-1) was performed. The fissure between the right upper and middle lobes was complete, whereas the fissure between the upper and lower lobes was incomplete. The latter was divided using the POWERED ECHELON FLEX GST system with 60 mm gold and white cartridges. LigaSure was used for division of the ascending A2 branch and upper mediastinal lymph node dissection but was not applied directly to the lung parenchyma. No apparent thermal or mechanical injury to the residual lung was observed. An intraoperative air-leak test under an airway pressure of approximately 15–20 cm H_2_O showed no air leak. The bronchial stump and staple line were reinforced with a NEOVEIL sheet and BOLHEAL. A 20-Fr chest tube was placed at the apex. No postoperative air leak was observed. The chest tube was removed on postoperative day 4 after confirmation of adequate lung expansion without subcutaneous emphysema. The postoperative course was uneventful, and the patient was discharged on postoperative day 5.

On postoperative day 25, the patient returned with a fever of 38.2°C, cough, and mild hemoptysis. His oxygen saturation was 98% on room air. Laboratory tests showed a white blood cell count of 11.0 × 10^9^/L, C-reactive protein level of 61.2 mg/L, and hemoglobin level of 120 g/L. Chest radiography demonstrated a large cavitary lesion with an air–fluid level in the right lower lung field (Fig. 1A). CT demonstrated a 173 × 104-mm thin-walled cavitary lesion with an air–fluid level above the right diaphragm and mild surrounding inflammatory changes (Fig. 1B). Bronchial stump failure with an infected pleural space was initially suspected, and a 20-Fr double-lumen trocar catheter was inserted at the bedside into the presumed infected cavity on the same day. Approximately 600 mL of dark-red, non-malodorous fluid was initially drained. No air leak was observed, and subsequent imaging demonstrated early reduction in the size of the cavitary lesion. Bronchoscopy performed on postoperative day 25 demonstrated a smooth, intact right upper-lobe bronchial stump without dehiscence, slit-like defect, necrosis, or purulent secretions, making bronchial stump failure unlikely. Culture of the drained fluid was negative for bacterial growth. Intravenous sulbactam/ampicillin (3 g every 6 h) was administered for 19 days. At discharge, antimicrobial therapy was transitioned to oral amoxicillin/clavulanate plus amoxicillin for 5 days. The middle and lower lobe bronchi were narrowed at their orifices by extrinsic compression, but the distal mucosa was normal. After drainage, imaging showed re-expansion of the residual lung and early shrinkage of the cavity (Fig. 1C), and inflammatory markers improved.

**Figure 1.**
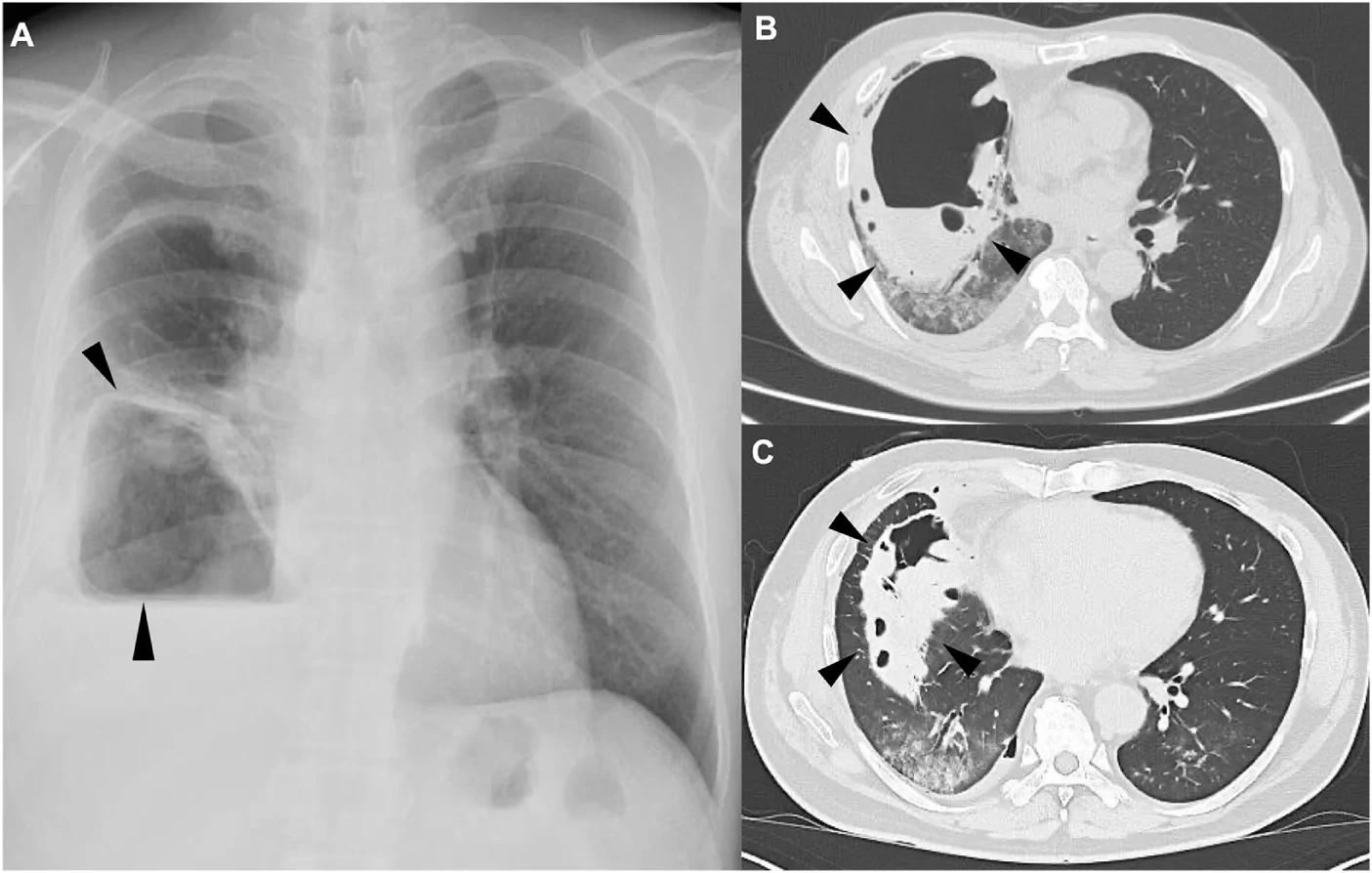
Imaging findings on postoperative day 25 and after drainage. (A) Chest radiograph showing a cavitary lesion with an air–fluid level (arrowhead). (B) Chest computed tomography showing a cavitary lesion with an air–fluid level, compression of the basal segmental bronchus, and inflammatory changes in the surrounding lung parenchyma (arrowhead). (C) Chest computed tomography after chest drainage showing a decrease in the size of the cavitary lesion (arrowhead).

Because the nature of the lesion remained uncertain, thoracoscopic exploration and drainage were performed under general anesthesia on postoperative day 36. The residual lung was extensively adherent to the chest wall, diaphragm, and mediastinum, and a small amount of dark-red pleural fluid was present dorsally. No frank empyema cavity or purulent coating was identified. A relatively thin-walled cystic lesion continuous with the right lower lobe was identified; however, the boundary between the cyst wall and normal lung parenchyma was indistinct (Fig. 2). Thoracoscopic inspection confirmed that the previously inserted 20-Fr drainage catheter was located within the cystic cavity and had therefore unintentionally decompressed the lesion. No spontaneous fistulous communication between the cyst and pleural cavity, and no intraoperative air leak, were identified. The tract associated with the initial catheter placement was considered iatrogenic rather than a spontaneous fistula. Cyst resection was not attempted because the boundary between the cyst and residual lung was indistinct and the residual lung was densely adherent to the chest wall, diaphragm, and mediastinum, making further dissection potentially hazardous. Based on the thin-walled cystic morphology, intact bronchial stump, absence of frank empyema, thoracoscopic findings, and subsequent radiographic regression, a clinical diagnosis of postoperative pneumatocele was made. Histopathological confirmation of the cyst wall was not obtained. The initial catheter remained in place until postoperative day 36, when it was removed during thoracoscopic exploration and replaced with a new 20-Fr double-lumen catheter within the cystic cavity for continued decompression. An additional 24-Fr double-lumen chest tube was placed in the pleural cavity. No postoperative air leak was observed, and drainage progressively decreased to 90 mL/day. After a 3-day clamp test confirmed no re-expansion of the cystic lesion on CT, the drains were removed on post-thoracoscopy day 6. The patient was discharged on post-thoracoscopy day 8 without fever, dyspnea, or oxygen requirement. Because thoracoscopic intervention under general anesthesia was required, the postoperative complication was classified as Clavien–Dindo grade IIIb. Follow-up CT demonstrated progressive regression of the lesion, with a marked decrease in size at 4 months (Fig. 3A) and near-complete resolution with only minimal residual scarring above the diaphragm at the latest imaging follow-up (Fig. 3B). No recurrence was observed.

**Figure 2.**
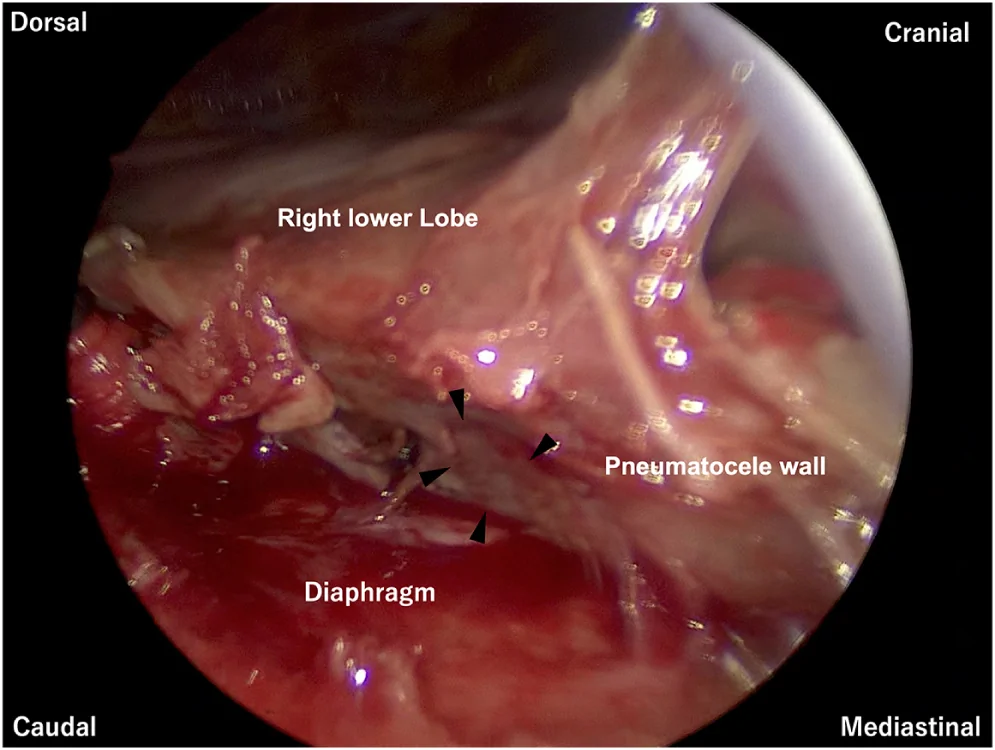
Thoracoscopic findings. (A) The right lower lobe was adherent to the diaphragm. (B) Part of the pneumatocele wall was identified on the lower lobe side (arrowhead).

**Figure 3.**
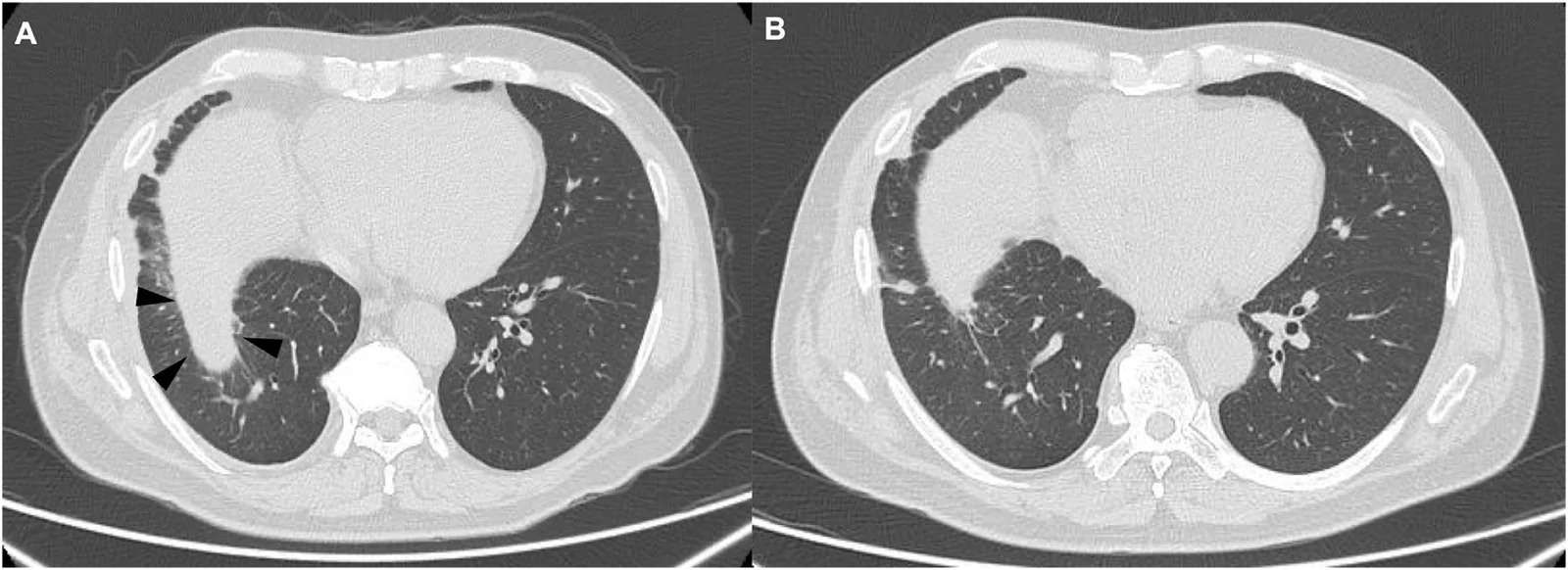
Follow-up chest computed tomography after treatment. (A) At 4 months after treatment, the lesion had decreased in size above the diaphragm. (B) Follow-up CT shows near-complete resolution of the lesion with minimal residual scarring above the diaphragm. CT, computed tomography.

## Timeline

Day 0 (13 February 2025): Video-assisted thoracoscopic right upper lobectomy with ND2a-1.

POD 4: The initial chest tube was removed.

POD 5: Discharged after an uneventful postoperative course.

POD 25: Readmission for fever, cough, and mild hemoptysis; CT showed a 173 × 104-mm cavitary lesion with an air–fluid level. Bronchoscopy and drainage were performed.

POD 36: Thoracoscopic exploration and drainage were performed.

POD 42: Drains were removed after a 3-day clamp test.

POD 44: Discharged without respiratory symptoms.

Follow-up: Progressive regression of the lesion without recurrence.

## Discussion

Postoperative pneumatoceles have been reported predominantly in the residual lung after upper lobectomy^[^2–5^]^. A plausible mechanism is that postoperative re-expansion and cranial relocation of the remaining lobes concentrate mechanical stress in a limited region, predisposing to localized overinflation^[^2^]^. In addition, a peripheral airway check-valve phenomenon may generate regional air trapping; superimposed postoperative intrathoracic pressure fluctuations and transient pressure loads from coughing can promote progressive cyst formation^[^6^]^.

Histopathologic evaluations in recent reports have suggested that the cyst wall may consist of visceral pleura and relatively preserved lung parenchyma without overt necrosis or infection^[^4,5^]^. These observations support a pathophysiology of visceral pleural detachment and progressive distension rather than parenchymal destruction or true abscess formation. Although histopathology was not obtained in our patient, the initial lobectomy was performed without pleural adhesions, parenchymal fragility, or excessive traction on the residual lung. Subsequent imaging did not demonstrate frank parenchymal necrosis, and culture of the drained fluid showed no bacterial growth, findings that were broadly consistent with the reported pathologic features.

Previously reported contributing factors include underlying lung vulnerability, prolonged postoperative air leak, and positive-pressure ventilation^[^2,3,6^]^. Our patient had no chronic obstructive pulmonary disease, postoperative air leak, or requirement for postoperative positive-pressure ventilation. The upper-to-lower interlobar fissure was incomplete and was divided with staplers; the energy device was not applied directly to the lung parenchyma, and no apparent thermal or mechanical injury was identified intraoperatively. Postoperative displacement of the residual lower lobe, local mechanical stress at the divided fissure, coughing, and a check-valve mechanism may have contributed to cyst formation. Although transient perioperative airway pressure could theoretically contribute, its relevance in the present case remains speculative because the patient was extubated immediately after surgery and did not require postoperative positive-pressure ventilation^[^2,3,6^]^.

The principal differential diagnoses in this case were bronchial stump failure, empyema, lung abscess, and postoperative pneumatocele. Bronchial stump failure with an infected pleural space was initially suspected because of the fever and large cavitary lesion with an air–fluid level. However, bronchoscopy demonstrated an intact bronchial stump without dehiscence or purulent secretions. Thoracoscopy subsequently did not reveal a frank empyema cavity and identified a relatively thin-walled cystic lesion that was continuous with the residual right lower lobe. Together with the subsequent radiographic regression after decompression, these findings supported a clinical diagnosis of postoperative pneumatocele. Nevertheless, because the cyst wall was not resected, histopathological confirmation was not available.

Importantly, the clinical course in our patient should not be interpreted as purely conservative management. The initially placed drainage catheter unintentionally entered the cystic cavity, resulting in evacuation of a substantial volume of fluid and early radiographic reduction of the lesion. Direct cyst decompression, therefore, likely contributed to the regression. Observation with antimicrobial therapy and serial imaging may be appropriate for clinically stable patients, whereas drainage or decompression may be considered for large, symptomatic, or progressively enlarging lesions. Fenestration or direct cyst drainage has been described in a limited number of case reports^[^4,5^]^; however, evidence supporting specific indications remains insufficient, and these procedures should be considered on an individual basis. Recognition of postoperative pneumatocele may help avoid unnecessary major procedures such as bronchial stump reconstruction or additional pulmonary resection, once bronchial stump failure and uncontrolled empyema have been adequately excluded.

This report has several limitations. First, histopathological confirmation of the cyst wall was not obtained; therefore, the diagnosis of postoperative pneumatocele was based on imaging, bronchoscopic, and thoracoscopic findings, and the subsequent clinical course. Second, the precise mechanism of cyst formation remains uncertain, and the possible contributions of perioperative airway pressure, postoperative lobe displacement, and local mechanical stress remain speculative. Finally, because direct cyst decompression occurred unintentionally during the initial drainage procedure, the independent contributions of drainage, antibiotic therapy, and spontaneous regression cannot be determined.

## Conclusion

We encountered a clinically diagnosed postoperative pneumatocele after right upper lobectomy. When a new cavitary lesion with an air–fluid level develops after pulmonary resection, bronchial stump failure and empyema should be carefully excluded. Bronchoscopy and thoracoscopy may facilitate differential diagnosis. In selected patients, cyst decompression or drainage followed by close observation may allow resolution without additional pulmonary resection.

## Data Availability

The data supporting the findings of this case report are available from the corresponding author upon reasonable request.

## Patient perspective

The patient reported that he was pleased that his cough and other respiratory symptoms had resolved during follow-up.

## Use of Artificial Intelligence

During the preparation and revision of this manuscript, the authors used ChatGPT (OpenAI) solely for English-language editing and refinement of phrasing. No identifiable patient information or clinical images were entered into the AI tool. All AI-assisted text was critically reviewed, fact-checked against the original clinical data, and edited by the authors, who take full responsibility for the accuracy and integrity of the final manuscript. AI was not used for clinical decision-making, data analysis, interpretation of results, or generation of figures.

## References

[1] Kerwan A Al-jabir A Mathew G. Revised Surgical CAse REport (SCARE) guideline: an update for the age of Artificial Intelligence. Prem J Sci 2025;10:100079.

[2] Sugimura A Takahashi T Sekihara K. Rapid formation of intraoperative pulmonary pneumatocele after lobectomy. Ann Thoracic Surg 2020;110:e331–e332. 10.1016/j.athoracsur.2020.03.028 32302662

[3] Kawamoto N Okita R Hayashi M. Rapid development and rupture of a pulmonary cyst in the early postoperative period after pulmonary resection. Thorac Cancer 2020;11:1712–15. 10.1111/1759-7714.13421 PMC7262938 32246895

[4] Okita R Okada M Kawamoto N. Rapid development and rupture of a pneumatocele caused by pulmonary dissection in the early postoperative period of lung resection: a case report. AME Case Rep 2022;6:25. 10.21037/acr-21-37 PMC8762382 35128314

[5] Tanaka T Sato M Shimizu K. Abrupt bulla formation by visceral pleural detachment after pulmonary lobectomy: a case report. J Chest Surg 2024;57:1–5. 10.5090/jcs.22.143 PMC10165434 36710575

[6] Kondo Y Nakao M Hashimoto K. Lower lobe pneumatoceles following upper lobectomy: report of two cases. Ann Thoracic Surg 2021;112:e403–e406. 10.1016/j.athoracsur.2021.02.081 33737042

